# Commentary on: SMARCB1 as a novel diagnostic and prognostic biomarker for osteosarcoma

**DOI:** 10.1042/BSR20220040

**Published:** 2022-06-09

**Authors:** Consolato M. Sergi

**Affiliations:** 1 AP Division/Pathology Laboratories, Children’s Hospital of Eastern Ontario, University of Ottawa, 401 Smyth Rd, Ottawa, Ontario K1H 8L1, Canada; 2 Department of Laboratory Medicine and Pathology, University of Alberta, Edmonton, AB, Canada; 3 Department of Orthopedics, Tianyou Hospital, Wuhan University of Science and Technology, Wuhan, Hubei, China

**Keywords:** gene expression and regulation, Osteosarcoma, Pathology

## Abstract

In the last couple of decades, biomarkers have been on the rise for diagnostic and predictive value. There has been a rush to identify new markers using new technologies and drug repurposing approaches. SMARCB1 acronym arises from the **S**WI/SNF (SWItch/Sucrose Non-Fermentable)-related **M**atrix-associated **A**ctin-dependent **R**egulator of **C**hromatin subfamily **B** member **1** (*SMARCB1*). It is a molecule, whose role is associated with the sucrose metabolism. *SMARCB1* is also called *INI1* (Integrase Interactor 1). The molecule was discovered in the mid-1990s. Its role as a loss-of-function marker for malignant rhabdoid tumors (MRT) of renal and extrarenal origin has enormously expanded the spectrum of involved neoplasms since that time. Several tumors have been characterized by genetic aberrations in the *SMARCB1* gene. They include reduction in expression, loss of expression, and mosaic expression. Most of the tumors are sarcomas, but a variegated group of tumors with mixed phenotypes has also been delineated. It is well known that the outcome of patients harboring genetic aberrations in the *SMARCB1* gene has been poor. Guo et al. reported that reduced *SMARCB1* expression occurred in 70% of osteosarcomas. Their data significantly correlated with poor neoadjuvant response. These authors emphasize a shorter progression-free and overall survival of the patients demonstrating an altered expression of this gene. Interestingly, mRNA *in silico* analysis established that *SMARCB1* expression correlates with the response to chemotherapy of osteosarcoma patients, but there was no reliable correlation between SMARCB1 expression level and metastasis, response to neoadjuvant therapy, overall survival, and progression-free survival. The study involved a tissue microarray (TMA) on bone tumors that may limit the full evaluation of the gene expression. Nevertheless, Guo et al.’s study is remarkable. It expands the list of the tumors harboring an altered *SMARCB1* gene expression and suggests that this marker should be investigated in every pathology workup for potential predictive value. On the other side, much work needs to be done if we hope that we strive to provide additional therapeutic strategies for osteosarcoma patients with altered *SMARCB1* gene expression.

SWI/SNF (SWItch/Sucrose Non-Fermentable)-related Matrix-linked Actin-dependent Regulator of Chromatin subfamily B member 1 (*SMARCB1*) expression has been a marker, increasing its spread in oncology and pathology as a diagnostic and predictive outcome marker in the last three decades. The gene is located on chromosome 22q11.23 and contains nine exons spanning approximately 50 kb. The *SMARCB1* gene is also called *INI1* (Integrase Interactor 1). It translates for a subunit of the SWI/SNF chromatin-remodeling complex, which is ATP-dependent [1–7].

In 2001, Turelli et al. demonstrated that incoming retroviral preintegration multiplexes intriguingly trigger the exportin-interceded cytoplasmic transfer of the SWI/SNF component INI1 and the nuclear body constituent PML (promyelocytic leukemia), a tumor-suppressor protein [8]. PML is essential for developing a macromolecular nuclear structure called the PML-associated nuclear body (PML-NB), which are often also called Kremer bodies, nuclear domains-10, and, of course, PML oncogenic domains. A few months later, Wu et al. identified that GADD34 and SNF5 can share a trimeric complex with chimeric fusion proteins [9]. It leads to inhibition of GADD34-arbitrated programmed cell death. The same authors suggested that GADD34 intercedes growth suppression through its interface with SNF5. A few years later, Vries et al. detected that loss of SNF5 function in malignant rhabdoid tumor (MRT)-originated cells headed to the instability of chromosomes and polyploidy [10]. Interestingly, the re-expression of SNF5 reinstated the coupling between cell cycle progression and ploidy checkpoints. In contrast, neoplasm-associated SNF5 mutations aggravated poly- and aneuploidy by abolishing chromosome segregation. Subsequently, loss of SNF5 function causes increased levels of MAD2 due to unregulated E2F1 activity, as identified by Vries et al. [10]. This results in causing a defective spindle checkpoint. They determined that SNF5 exerts ploidy control over a pathway that comprises cyclin D, CDK4, E2F, p16 (INK4a; CDKN2A), and RB1. Jagani et al. established that SNF5 is a negative controller of GLI-hedgehog signaling [11]. A few years ago, Wang et al. established that SMARCB1 increased the number of SWI-SNF complexes by experiments leading to the re-expression of SMARCB1 in brain and kidney rhabdoid cell lines and in Smarcb1-null mouse embryonic fibroblasts [12]. They also found increased protein levels of SWI/SNF subunits, predominantly ARID1A and ARID1B [13]. Chromatin immunoprecipitation analysis was key in determining that re-expression of SMARCB1 also augmented chromatin habitation by SMARCA4 and SMARCC1 [14–16]. SWI/SNF complexes chiefly targeted enhancers located distally to transcriptional start sites, no matter SMARCB1 was present or not. It also leads to the conclusion that SMARCB1 let stabilize SWI/SNF complexes at enhancers. Moreover, SMARCB1 has also been demonstrated to play a role in the down-regulation of the Wnt signaling pathway gene expression. It represses retinoblastoma (RB) target genes, including E2F factors and CCND1, promoting c-Avian Myelocytomatosis Viral Oncogene Homolog Hemoglobin (MYC) oncogene-mediated transactivation. In addition, it induces aberrant activation of hedgehog signaling by networking GLI1 and concentrating at GLI1-regulated promoters [11]. It also epigenetically influences the action of the polycomb complex.

Loss of *SMARCB1/INI1* expression is characteristically found in atypical teratoid/rhabdoid tumor of the central nervous system [17], renal rhabdoid tumors (pediatric and adult), and renal medullary carcinoma [18]. The list grew and it now includes epithelioid malignant peripheral nerve sheath tumor or malignant schwannoma [19], sino-nasal carcinoma [20,21], epithelioid sarcoma [22], extra-skeletal myxoid chondrosarcomas [23], pediatric poorly differentiated chordoma [24], and myoepithelial carcinoma [5,25–28]. Thus, both mesenchymal and epithelial malignant neoplasms are characterized by a genetic aberration of this key component of the chromatin arrangement.

Recently, Guo et al. investigated *SMARCB1* gene expression in osteosarcoma and its clinical significance with regard to chemotherapeutic sensitivity and outcome of their patients [29]. They examined 114 specimens from 70 osteosarcoma patients to build a tissue microarray (TMA) and assess the staining of SMARCB1 protein using a straightforward Avidin–Biotin Complex-based immunohistochemically run assay or immunofluorescence on paraffin blocks. The mRNA expression of this gene was investigated *in silico* using open-access RNA sequencing. They found a weak *SMARCB1* expression in about two-thirds of the osteosarcoma specimens in the TMA. Their data significantly correlated with poor neoadjuvant response and shorter progression-free and overall survival. The study of the mRNA by *in silico* analysis confirmed that *SMARCB1* expression correlates somehow with the response to chemotherapy and prognosis in osteosarcoma patients. However, it seems that there was no reliable correlation between SMARCB1 staining and metastasis, response to neoadjuvant therapy, overall survival, and progression-free survival. The reliability would be improved by detailed analysis of *SMARCB1* expression in different types of osteosarcomas.

The primary concern of the present study is the use of TMA [30]. Not to be misunderstood, TMA is terrific and beneficial to research, but limitations may apply and should be taken into account [31]. In Figure 1, a composite of three microphotographs of an osteosarcoma with variable *SMARCB1/INI1* expression is presented. To construct the TMA, the authors studied 114 formalin-fixed, paraffin-embedded (FFPE) osteosarcoma tissue blocks obtained from 70 enrolled patients. TMA has been a cost-effective choice for most pathology institutions and research groups. Still, the limited distribution of staining to small cores instead of the whole section has raised doubts on the validity of studies involving microarrays. Over the years, it has been concluded that an important and satisfactory number of cores needs to be present in the TMA block to be relevant. There is probably no safe rule because it also depends on the epitopes and on the retrieval techniques adopted. One major consideration is that the fixation of some of the bony tissue specimens relies on multiple hours of immersion with formaldehyde and subsequent decalcification, which may hamper the reproducibility of some data. Nevertheless, the study raises important aspects, and the increased number of cores used by Guo et al. in their study may consolidate the conviction that further studies are necessary to evaluate properly *SMARCB1* expression with the response to chemotherapy and prognosis in osteosarcoma patients. Children’s Oncology Group biorepositories and International Society of Pediatric Oncology (SIOP)’s databanks may be key for the research to further concretize the role of *SMARCB1/INI1* in future protocols of the College of American Pathologists [21,32].

**Figure 1:**
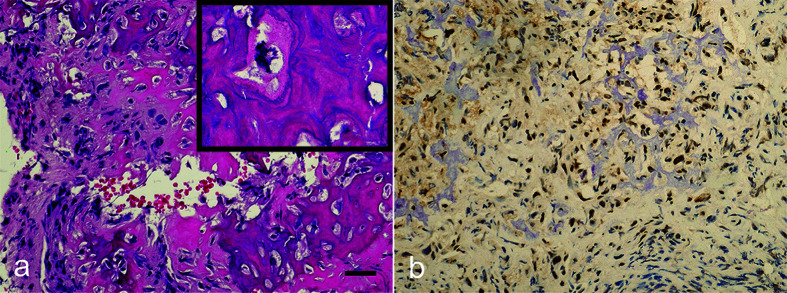
Osteogenic Sarcoma (Osteosarcoma) and SMARCB1 Expression (**a**) Microphotograph of an osteosarcoma showing osteoid production and anaplastic tumor cells. Bar is 20 μm. Anaplasia, which derives from the Greek words άνά (backward) and πλάσις (formation), is a biological condition of cells exhibiting poor cellular differentiation, loss of the morphological features of maturity, and poor orientation with respect to each other. Anaplastic cells demonstrate nuclear pleomorphism, abnormal nucleus-cytoplasm ratio, prominence of nucleoli, and increased proliferation index. In the microphotograph inset, a multipolar mitosis of a neoplastic cell (hematoxylin and eosin staining, 200x original magnification) is seen. (**b)** Microphotograph of an osteosarcoma showing variable nuclear staining for SMARCB1/INI1 (anti-SMARCB1/INI1 immunohistochemistry on formalin-fixed and paraffin-embedded tissue, Avidin–Biotin Complex, 200x original magnification).

The cumulative data on the activity of SMARCB1 in epigenetic regulation have provided an ideal platform to trial biologically targeted therapies. Currently, some agents include: (1) EZH2 inhibitors, which prevent *SMARCB1/INI1*-deficient neoplasms progression and have been proved to sensitize neoplastic cells to the effects of radiation therapy; (2) CDK4 inhibitors, which inhibit tumor cell growth by G1 arrest; (3) aurora-A-kinase inhibitors, inhibits aurora A; (4) histone deacetylase inhibitors (HDACi), with a similar mechanism of action to the EZH2 inhibitors; and (5) DNA methyltransferase inhibitors [33,34].

Overall, although the study involved a TMA, which may limit the full evaluation of the gene expression, Guo et al.’s study expand the list of the tumors harboring an altered *SMARCB1* gene expression and suggests that this marker should be investigated in every pathology workup for potential predictive value [29]. On the other side, much work needs to be done if we hope to provide additional therapeutic strategies for patients affected with osteosarcoma with or without altered *SMARCB1* gene expression [35–42].

## Abbreviations

FFPE: formalin-fixed, paraffin-embedded
INI1: Integrase Interactor 1
MRT: malignant rhabdoid tumors
NB: nuclear body
PML: pro-myelocytic leukemia
RB: retinoblastoma
SMARCB1: SWI/SNF-related Matrix-associated Actin-dependent Regulator of Chromatin subfamily B member 1
SWI/SNF: SWItch/Sucrose Non-Fermentable
TMA: tissue microarray
